# Wafer Defect Detection Technology Based on CTM-IYOLOv10 Network

**DOI:** 10.3390/jimaging11110408

**Published:** 2025-11-12

**Authors:** Pengcheng Ji, Zhenzhi He, Weiwei Yang, Jiawei Du, Guo Ye, Xiangning Lu

**Affiliations:** 1 School of Mechanical and Electrical Engineering, Jiangsu Normal University, Xuzhou 221116, China; 2 School of Mechanical and Electrical Engineering, China University of Mining and Technology, Xuzhou 221116, China

**Keywords:** wafer defect detection, YOLOv10, clustering–template matching, small object detection, real-time industrial inspection, intelligent manufacturing

## Abstract

The continuous scaling of semiconductor devices has increased the density and complexity of wafer dies, making precise and efficient defect detection a critical task for intelligent manufacturing. Traditional manual or semi-automated inspection approaches are often inefficient, error-prone, and susceptible to missed or false detections, particularly for small or irregular defects. This study presents a wafer defect detection framework that integrates clustering–template matching (CTM) with an improved YOLOv10 network (CTM-IYOLOv10). The CTM strategy enhances die segmentation efficiency and mitigates redundant matching in multi-die fields of view, while the introduction of a modified GhostConv module and an enhanced BiFPN structure strengthens feature representation, reduces computational redundancy, and improves small-object detection. Furthermore, data augmentation strategies are employed to improve robustness and generalization. Experimental evaluations demonstrate that CTM-IYOLOv10 achieves a detection accuracy of 98.1%, reduces inference time by 23.2%, and compresses model size by 52.3% compared with baseline YOLOv10, and consistently outperforms representative detectors such as YOLOv5 and YOLOv8. These results highlight both the methodological contributions of the proposed architecture and its practical significance for real-time wafer defect inspection in semiconductor manufacturing.

## 1. Introduction

With the design of semiconductor components for integration, an increasing number of integrated circuit components are being etched onto semiconductor wafers. It is widely used in information and communication, automotive, and aerospace applications. To make electronic products portable and multifunctional, chips must be smaller and tighter, which makes it necessary to accommodate more components on the wafer surface [1,2]. During the manufacturing process, cutting wafers can cause chipping and breakage of dies, and dust particles present in the clean room can also cause damage to the dies. However, the defects that appear on the wafer surface during the fabrication process of these wafers will seriously affect the wafer product quality and cause huge economic losses. Therefore, it is essential to study the problem of identifying defects on the wafer surface and adjusting the production line in time to improve the manufacturing yield [3,4].

To detect surface defects on wafers, the traditional inspection method is manual inspection, which has a low sampling rate and poor real-time performance, and is highly influenced by experience and subjective factors [5]. In the face of irregularly shaped and weakly imaged defects, traditional algorithms suffer from low performance, high false detection rates, and high noise sensitivity. Computer vision is more effective in detecting defects such as wafer stains, collapses, and cracks, which typically arise from processes such as lithography misalignment, particle contamination, or dicing stress during wafer fabrication [6]. The use of computer vision significantly reduces labor costs and is more suitable for highly integrated wafers. Therefore, some researchers have used deep learning to automatically identify features of interest in images [7,8,9]. Algorithms based on the neural network frameworks of the YOLO series exhibit good performance in terms of detection speed.

Compared to YOLOv5 and YOLOv8 [10,11,12,13], YOLOv5 introduces improved feature extraction modules and enhanced performance on small-scale datasets, facilitating its application in industrial defect detection. YOLOv8 further incorporates structural optimizations and refined training strategies, offering excellent detection accuracy and inference speed. These enhancements have enabled YOLO-based methods to achieve promising results in semiconductor defect detection.

To further advance YOLO-based defect detection, several studies have focused on wafer inspection tasks. Ma et al. [14] proposed the CC-De-YOLO framework, which embeds coordinate attention and a CAR-EVC upsampling module into YOLOv7 to better capture positional semantics for complex wafer defects. Similarly, Li et al. [15] developed an improved YOLOv7-tiny with attention modules and optimized loss functions, showing notable accuracy gains while keeping the model lightweight and efficient for real-time wafer inspection. More recently, Chi et al. [16] proposed CISC-YOLO based on the YOLOv8 framework, introducing lightweight modules and cross-scale fusion to achieve high performance on wafer defects with reduced model size and latency. These works demonstrate the adaptability of different YOLO versions in wafer defect detection and motivate our efforts to extend YOLOv10 for further improvements.

Building on this, YOLOv10 incorporates architectural optimizations, including an enhanced Bidirectional Feature Pyramid Network (BiFPN) neck, decoupled detection head, and lightweight backbone, leading to improvements in both accuracy and computational efficiency. Recent studies have explored YOLOv10’s potential in diverse scenarios. Zhang et al. [17] proposed UK-YOLOv10 with an attention-based backbone for surgical tool detection, whereas Sun et al. [18] introduced SOD-YOLOv10 to enhance small object detection in remote sensing images via transformer-based modules. Gao et al. [19] designed LAO-YOLO for drone-based lightweight detection, and Ji and Ma integrated YOLOv10 into a DETR-based vehicle-detection framework, achieving superior performance in complex traffic environments [20].

To solve the problem of classifying die defects inside wafers with high quality and efficiency, the improved template matching algorithm and the improved YOLOv10 algorithm are proposed in this paper [21]. When only one die is included in the field of view, the process of inspecting and then moving the camera to the next single die wastes a lot of time and affects the efficiency of inspecting the whole wafer. Therefore, multiple die objects appearing in the same field of view are used to improve the inspection efficiency. However, the channel of multiple die images can cause incorrect detection of wafer-surface defects. Therefore, a suitable template-matching algorithm was used to match each die in the image. In addition, to ensure that only one target object is matched for each die region, a clustering algorithm is introduced in combination with the template-matching algorithm [22].

In the improved algorithm, the region with the highest confidence level was selected and used for defect region detection in the YOLOv10 target detection model. Although the original YOLOv10 detection model demonstrated high accuracy and real-time performance, its computational cost and model size still pose challenges for deployment on resource-constrained platforms. To address this issue, lightweight convolutional modules, such as GhostNet [23], were incorporated into the backbone of YOLOv10 to reduce redundant computations and significantly compress the model parameters. In contrast to traditional convolution, GhostNet enables efficient feature extraction with lower memory overhead, facilitating mobile deployment. Furthermore, to enhance the recognition capability for small targets, the BiFPN is utilized as the neck structure, replacing earlier FPN-based designs and enabling more efficient multi-scale feature aggregation without increasing computational complexity.

Liu et al. [24] integrated attention mechanisms and a lightweight detection head into YOLOv10 to improve the detection accuracy of tiny tomato fruits in industrial sorting tasks. Zhu et al. [25] proposed YOLO-HPSD, which combines BiFPN with a mixed local channel attention module to enhance feature representation across scales, thereby achieving high-precision ship detection in maritime environments.

Although YOLOv10 and its derivatives have demonstrated promising results in natural image tasks and general object detection, their application in semiconductor wafer defect inspection remains unexplored. In this context, we propose a customized approach that integrates clustering–template matching with an improved YOLOv10 framework, aiming not only to enhance detection accuracy but also to improve robustness under varying defect shapes, sizes, and imaging conditions. This study introduces an end-to-end detection system specifically designed for wafer-level inspection, offering a practical solution that addresses both algorithmic efficiency and real-world deployment challenges.

In this study, a wafer defect dataset including the collapse, cut-off, and stain categories was constructed [26]. Building upon this dataset, we propose a defect detection method that integrates clustering–template matching with an improved YOLOv10 network. The clustering–template matching algorithm enhances die segmentation efficiency, while GhostConv modules and an enhanced BiFPN structure improve model compactness and small-target recognition. Experimental results demonstrate that the proposed CTM-IYOLOv10 achieves higher accuracy, reduced model size, and faster inference, thereby effectively overcoming the limitations of manual wafer inspection, such as low efficiency, missed detections, and insufficient real-time performance.

## 2. The Improved Yolov10-Ctm Algorithm

**Figure 1:**
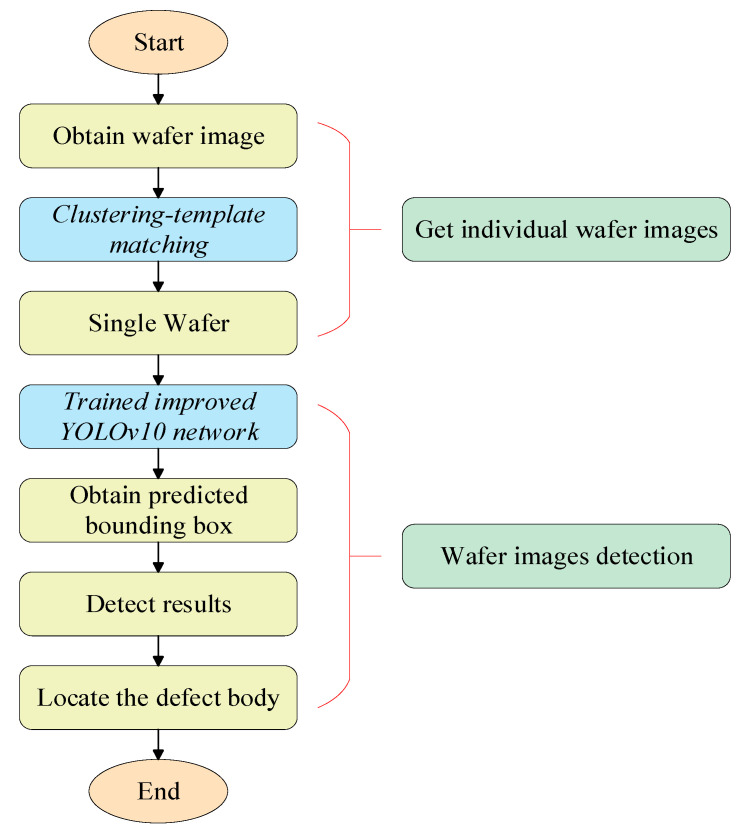
Schematic diagram of the real-time wafer defect detection system based on the improved YOLOv10.

In this study, the developed inspection platform and software were used to acquire wafer images in real-time. The acquired wafer images required grayscale processing to simplify the matrix and increase the runtime speed. The grayscale image is then matched with a clustering template, which is used to locate each die in the field of view. Finally, the improved YOLOv10 network structure was used to detect defects on the wafer surface and locate defective parts. A schematic of the real-time wafer inspection system based on the clustering template matching algorithm and the improved YOLOv10 network structure is shown in Figure 1.

### 2.1. Clustering–Template Matching Algorithm

**Figure 2:**
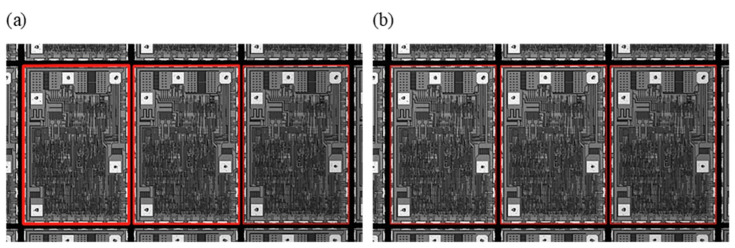
Match box results. (**a**) Original template matching; (**b**) Clustering–template matching.

During wafer inspection, multiple dies are contained in a single field of view. Channels between wafers can affect the accuracy and precision of the defect detection. Therefore, a suitable algorithm was selected and used to match all die images. A template-matching algorithm was used to satisfy the practical requirements of wafer inspection. The principle is to use template wafer image to slide over the field-of-view image. The similarity between the template image and corresponding image region was calculated, and the region with the highest similarity was selected for matching. In this study, the normalized inverse method was used for the similarity calculation, as shown in Equation (1).

(1) $$R(i,j)=\frac{\sum_{m=1}^{M}\sum_{n=1}^{M}\left[S^{i,j}(m,n)T(m,n)\right]}{{\left\{\sum_{m=1}^{M}\sum_{n=1}^{M}{\left[S^{i,j}(i,j)\right]}^{2}\right\}}^{1/2}\times {\left\{\sum_{m=1}^{M}\sum_{n=1}^{M}{\left[T(i,j)\right]}^{2}\right\}}^{1/2}}$$

where R(i,j) indicates the similarity between the template T and $S^{i,j}$. A larger R(i,j) value indicates a higher correlation between the images. When R(i,j) is larger than a certain threshold, the images are matched. To ensure that all dies were matched successfully, multiple matching results were obtained by setting the filtering threshold. However, the results of multiple redundant matching are obtained at the end of image matching, as shown in Figure 2a.

To solve the multi-objective matching redundancy problem while ensuring that all complete dies in the field of view can be matched, a clustering algorithm is used to compute and obtain the number of regions to be matched autonomously. Therefore, the Affinity Propagation (AP) algorithm, which does not require a specified number of clusters, was used to implement the partitioning of dies. The one with the highest confidence level in each region is selected, which guarantees that only one optimal result is matched in each region, as shown in Figure 2b.

The basic idea of the Affinity Propagation algorithm considers all samples as nodes of the network, and then calculates the clustering center of each sample through the message passing of each edge in the network. By continuously iterating the attractiveness r(i,j) and attribution, a(i,j) produces m high-quality prime centers, whereas the remaining data points are assigned to the corresponding clusters, as shown in Equations (2) and (3).

The basic idea of the AP algorithm is to treat all samples as nodes in a network [27], and to calculate the cluster center of each sample through message passing between these nodes. In this process, the algorithm iteratively updates two types of messages: the responsibility r(i,j), which reflects how well-suited sample j is to serve as the exemplar for sample i, and the availability a(i,j), which indicates how appropriate it would be for sample i to choose sample j as its exemplar. Through these updates, the algorithm identifies high-quality exemplar points and assigns the remaining data points to the corresponding clusters. The update rules are provided in Equations (2) and (3).

(2) $$r(i,j)=s(i,j)-\underset{j^{\prime }\neq j}{\max}\left\{a\left(i,j^{\prime }\right)+s\left(i,j^{\prime }\right)\right\}$$

(3) $$a(i,j)=\left\{\begin{array}{c} \min\left\{0,r(j,j)+\sum_{i^{\prime }\notin \{i,j\}}\max\left(0,r\left(i^{\prime },j\right)\right)\right\},i\neq j\\ \sum_{i^{\prime }\neq j}\max\left(0,r\left(i^{\prime },j\right)\right),i=j \end{array}\right.$$

In these equations, the similarity measure s(i,j) represents the negative squared Euclidean distance between data points i and j. The responsibility r(i,j) quantifies how well-suited point j is to serve as the exemplar for point i, while the availability a(i,j) reflects the accumulated evidence for point i to choose point j as its exemplar. These two messages are updated iteratively by the AP algorithm until convergence, enabling the selection of representative exemplars for clustering. As illustrated in Figure 2b, this process guarantees a unique matching result for each wafer region. The matching performance before and after applying the clustering algorithm is summarized in Table 1.

### 2.2. Model of Improved Yolov10 Network

YOLOv10 is the latest iteration in the YOLO object detection series that achieves a new balance between speed and accuracy. It introduces an efficient decoupled head design and integrates a lightweight backbone to significantly reduce the computational cost without sacrificing the detection precision. YOLOv10 includes various model scales to satisfy different deployment requirements, from edge devices to high-performance servers. With advanced architectural optimizations and training strategies, YOLOv10 achieved state-of-the-art performance on COCO and other benchmark datasets. The model also supports automatic anchor-free detection and offers native compatibility with ONNX version 16, TensorRT version 8.6, and CoreML version 6.3, thereby enabling rapid deployment across platforms. Building on the PyTorch framework version 2.2, YOLOv10 ensures easy customization, efficient training, and smooth conversion to various inference formats, making it suitable for industrial, mobile, and embedded AI applications.

**Figure 3:**
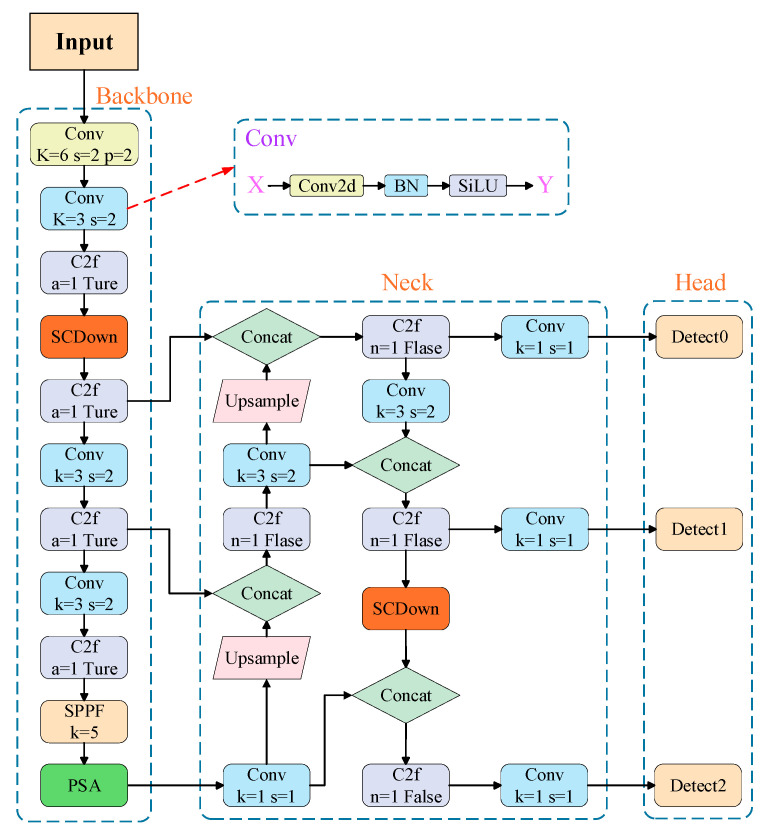
YOLOv10 network structure diagram.

In this study, YOLOv10 was used for wafer defect detection. The framework structure of YOLOv10 consists of four main parts: the Input, Backbone, Neck, and prediction head. The Input section enriches the dataset through a data augmentation method that adopts a lightweight and efficient design composed of Conv, C2f, and SPPF modules to extract rich semantic and spatial features from the input images. Neck employed an improved BiFPN structure for efficient multiscale feature fusion, enabling better localization and classification of defects at different scales. The Head section makes target predictions and outputs them through predictions. The overall architecture of the YOLOv10 network is illustrated in Figure 3.

#### 2.2.1. The Improved Yolov10 Network Structure

**Figure 4:**
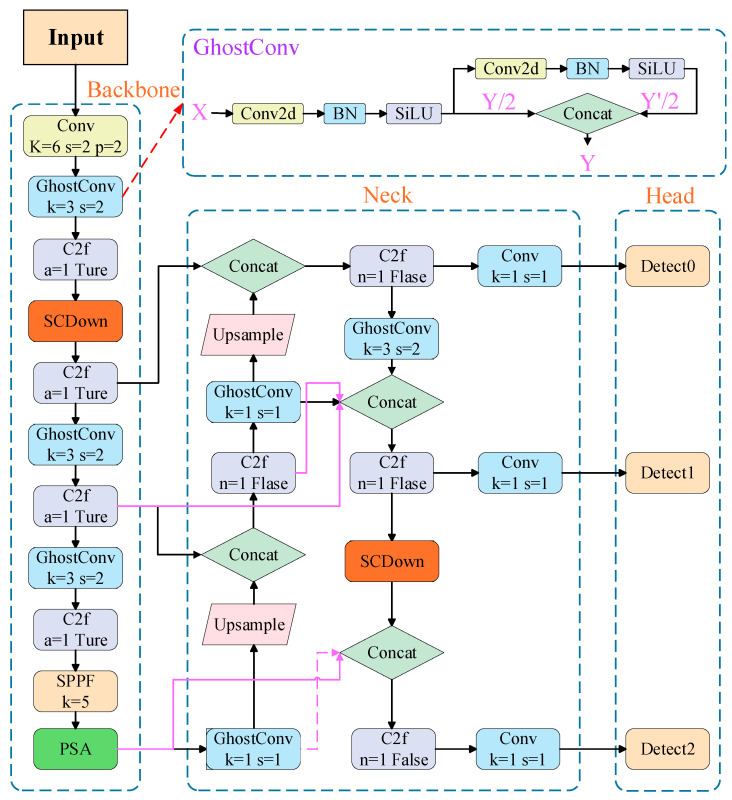
Improved YOLOv10 network structure diagram.

When the YOLOv10 network model is applied to detect wafers, the redundant parameter computation and longer training elapsed time affect not only the model training efficiency and size but also the detection speed. To solve this problem, the Conv module was replaced by a modified GhostConv module, which reduced the computational effort of the network and the weight of the model, and improved the detection speed. A lightweight model was obtained using this improvement. The second problem is that lightweight models do not significantly improve the detection accuracy of small target defects. It often misses notches at die edges. To improve the detection accuracy of small objects, YOLOv10 adopts an enhanced BiFPN structure in the neck section by design, which facilitates more effective multiscale feature fusion. Meanwhile, a data enhancement strategy is added to the input, which enhances the robustness of the model. The structure of the improved YOLOv10 network is illustrated in Figure 4.

**Figure 5:**
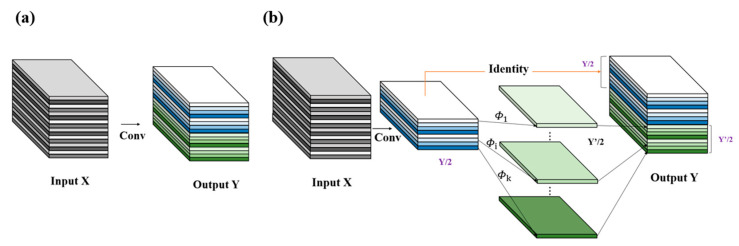
Convolutional layer. (**a**) Conv; (**b**) GhostConv.

Excellent target-detection networks are often computationally heavy in terms of parameters, and accelerating target-detection algorithms to meet the requirements of industrial applications is a pressing problem. Two common approaches have been used to achieve model acceleration: lightweight model design and network pruning. A new lightweight network, GhostNet [23], found three pairs of extremely similar feature maps, and thus these feature map pairs were considered redundant with each other. However, redundant information may be an important component of this model. Therefore, instead of attempting to remove these redundant feature maps, a lower computational effort was used to obtain them. The final feature set was generated by combining $Y^{\prime }/2$ and Y/2, effectively reducing the computational effort of the convolutional layer with little performance loss. This idea was introduced into the YOLOv10 network by improving the Conv module into the GhostConv module, which makes the model lighter and reduces the model computational overhead. The original convolutional layer and the improved GhostConv are shown in Figure 5.

To further enhance the network’s efficiency and feature representation, the standard three-by-three convolutional layers in the first three stages of the backbone were replaced with GhostConv modules. This modification retains the original stride and output dimensions while reducing redundant feature generation through the reuse of intrinsic feature correlations. Convolutional layers with a stride of two maintain the spatial structure while requiring fewer computations, effectively reducing parameters and floating-point operations without compromising learning capacity. This optimization lays the foundation for subsequent improvements in feature fusion.

**Figure 6:**
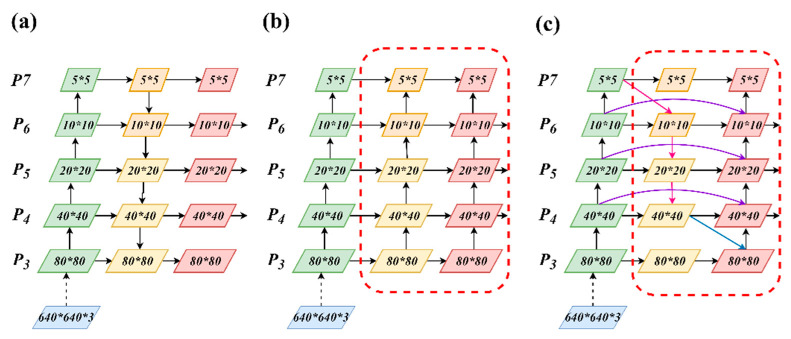
Feature fusion methods. (**a**) FPN; (**b**) FPN + PANet; (**c**) BiFPN.

Building on this design, the PAN structure in YOLOv10 was replaced with a more flexible BiFPN architecture. This structure introduces weighted bidirectional connections, additional cross-scale pathways, and unified channel-wise fusion, improving the balance of feature contributions across multiple resolutions. These enhancements strengthen semantic aggregation and are particularly beneficial for detecting small targets, which often suffer from weak feature representation in deep convolutional layers. The BiFPN fuses features from P3 to P7 through repeated bidirectional cross-scale connections, where each node receives inputs from both top-down and bottom-up paths, and fusion weights are adaptively learned during training through a normalized softmax mechanism to ensure stability. Figure 6 illustrates the structural evolution from FPN to PANet and BiFPN, highlighting the transition from unidirectional to weighted cross-scale fusion.

In modern target-detection frameworks, features of large objects with more pixels are easily retained, whereas those of small objects are often lost during deep convolution. The analysis revealed that after multiple convolution processes, the final convolution result is typically used for prediction; therefore, small targets are frequently underrepresented. To address this limitation, the FPN structure was first introduced in YOLOv3 to enhance multi-scale fusion [28]. The traditional FPN structure performs upsampling after multiple downsampling steps and fuses features at the same scale, forming the foundation for multi-scale representation, as illustrated in Figure 6a. After multiple downsampling and upsampling operations, feature layers at the same scale overlap, allowing three detection layers at different scales to integrate target information. To further improve performance, YOLOv4 and YOLOv5 adopted the FPN + PANet structure [29], as shown in Figure 6b, which enhanced bidirectional fusion but introduced unbalanced contributions between input features of different resolutions.

**Figure 7:**
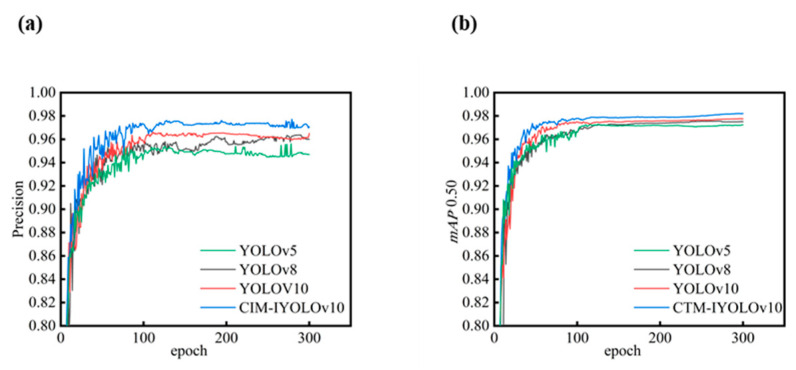
Comparison Metrics. (**a**) precision; (**b**) *mAP*0.50.

In the proposed algorithm, BiFPN is adopted as the core feature fusion structure in the YOLOv10 framework to enhance multi-scale representation and improve the detection of small targets [30]. BiFPN introduces a fast normalized fusion algorithm with learnable weights, which adaptively assigns importance to the input features across different layers. This weighted fusion mechanism allows the network to focus on informative features, while reducing redundant node connections. Additionally, the BiFPN incorporates extra input links when the input and output nodes share the same resolution, facilitating richer information exchange across layers. These enhancements improve the model’s capability in multiscale target recognition without increasing computational complexity. The overall structure of the BiFPN is illustrated in Figure 6c, and the fusion algorithm is formulated in Equations (4) and (5).

(4) $$P_{i}^{td}=Conv\left(\frac{\omega_{1}\times P_{i}^{in}+\omega_{2}\times Resize\left(P_{i+1}^{in}\right)}{\omega_{1}+\omega_{2}+\epsilon }\right)$$

(5) $$P_{i}^{out}=Conv\left(\frac{\omega_{1}^{\prime }\times P_{i}^{in}+\omega_{2}^{\prime }\times P_{i}^{td}+\omega_{3}^{\prime }\times Resize\left(P_{i-1}^{in}\right)}{\omega_{1}+\omega_{2}+\omega_{3}+c}\right)$$

where $P_{i}^{td}$ is the intermediate feature at level i on the top-down pathway, and $P_{i}^{out}$ is the output feature at level i on the bottom-up pathway. All features were constructed in a similar manner. Precision and mean Average Precision (*mAP*) are used as evaluation metrics to assess the model, which are defined in Equations (6) and (7).

(6) $$mAP=\frac{1}{N}\sum_{i=1}^{N}AP_{i}$$

(7) $$mAP=\frac{1}{N}\sum_{i=1}^{N}AP_{i}$$

where TP, FP, and FN represent the numbers of true positives, false positives, and false negatives, respectively. Based on these values, precision and *mAP* are adopted as the main performance metrics, as defined in Equations (6) and (7). The *mAP* metric was used to reflect the overall detection performance across the different confidence thresholds. The detection performances of the baseline YOLOv10 and the proposed CTM-IYOLOv10 were compared using these metrics. As shown in Figure 7a,b, the precision and *mAP*0.50 of CTM-IYOLOv10 exhibited significant improvements over the original model, indicating a lower miss rate and enhanced robustness. The improved model was capable of identifying more small defect targets within the same image, verifying its effectiveness in complex inspection scenarios.

To achieve both high recall and precise localization, the proposed CTM-IYOLOv10 framework integrates the improved YOLOv10 model with a CTM module in a cascaded manner. Specifically, the improved YOLOv10 network is responsible for coarse defect region localization by generating initial bounding boxes. These predicted regions are then passed as Region-of-Interest (ROI) inputs to the CTM module, which performs refined defect positioning based on structural similarity and contour features. This sequential pipeline enables fast candidate generation through YOLOv10 and accurate refinement through CTM, especially improving the localization of small or edge-near defects. The integration is modular, requiring no alteration to the YOLOv10 backbone or detection head, and is implemented as a post-processing step after bounding box prediction.

#### 2.2.2. Data Augmentation

Deep learning has high requirements for datasets in the sense that the size and sample diversity of the dataset influence the generalization ability of the training model. However, large-scale wafer datasets are created, which requires a significant amount of time. Data Augmentation is an effective way to expand the data size and improve the detection performance. Multiple data enhancement strategies were chosen, which can increase the number of training datasets and enrich the diversity of training datasets while improving the robustness and generalization of detection models [31]. In this study, some effective data processing methods, including image scrambling, brightness, contrast, saturation, hue, added noise, random scaling, random cropping, and flipping, were added to YOLOv10. These effective data processing methods can improve the accuracy of the training model and reduce training time.

Moreover, a hybrid data enhancement method, that is, copy–paste, was added to the defect detection of wafers. It copies and pastes the target from one sample to another, which increases the richness of the dataset without changing its size. A mosaic process was also added, which significantly enriched the background of the images. Four images were randomly cropped and stitched together into one image as training data [32]. However, stitching the four images together inevitably increases the batch size. Therefore, it is necessary to compute the batch normalization for the four images. The combination of copy–paste, mosaic, and traditional data enhancement strategies greatly improves the performance of target detection in a simple and effective manner.

## 3. Results and Discussion

### 3.1. Dataset Preparation and Experimental Configuration

A machine-vision inspection platform was used to photograph the wafers. A total of 2000 defect images containing stains, collapses, and cutoffs were acquired by collecting and data-enhancing, which constituted the wafer dataset. Then, each image in the dataset containing one or more defects was manually labeled using Labeling software. The specific compositions of these datasets are listed in Table 2.

The dataset was divided into training, validation, and testing sets in a ratio of 7:2:1. To ensure robustness, all experiments were repeated three times without applying cross-validation. Model training and inference were performed in a standardized computational environment. The detailed hardware and software configurations are summarized in Table 3, and the key training hyperparameters are listed in Table 4. Real-time detection in this study refers to the model’s ability to perform inference with a latency of less than 20 milliseconds per image, enabling efficient and continuous wafer inspection during production.

### 3.2. Detection Results

In the experiment, YOLOv10 without template matching and improved CTM-IYOLOv10 were used to detect the same die images. In the detection results, the correct recognition rate, false detection rate, and missed detection rate are important evaluation metrics that are used for comparative analysis. Below is a comparison between CTM-IYOLOv10 and YOLOv10 in a real detection scenario.

**Figure 8:**
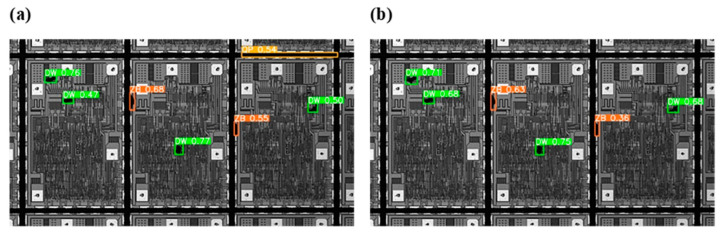
Defect detection results. (**a**) Defective areas detected without template matching; (**b**) Defective areas detected with the template matching.

In Figure 8a, it can be observed that before adding the clustering template matching algorithm, the YOLOv10 target detection model detected seven defects, including two collapse (ZB) defects, four stain (DW) defects, and one cutoff (QP) defect. Although all defect areas were detected, the two cutoff defects were incorrectly identified owing to the wafer channel. This false detection can be mainly attributed to the high visual similarity between the wafer edge grooves and actual cutoff defects. Without clustering and template-based die localization, the model lacks spatial priors to distinguish these features, resulting in misclassification in structurally complex channel regions. In contrast, the improved YOLOv10 detection model adds clustering–template matching. By matching dies individually, the problem of false detection in the channel regions is solved. In Figure 8b, all defects of the three complete dies in the image are accurately detected. Comparing the detection results, the improved algorithm assigns a higher confidence level to the defect regions, which facilitates the detection of various defects in dies.

**Figure 9:**
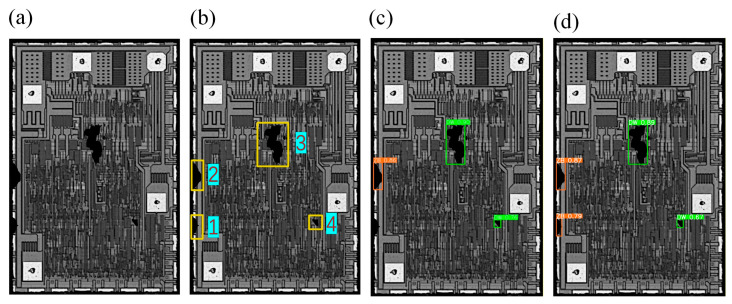
Defect detection results. (**a**) Original image; (**b**) Defective areas expected to be detected; (**c**) Defective areas detected by the YOLOv10 algorithm; (**d**) Defective areas detected by the improved YOLOv10 algorithm.

In Figure 9, there are four defective regions in the die image, including two stain defects and two edge collapse defects, as shown in Figure 9b. Both the original YOLOv10 model and improved model showed no detection errors. However, the original YOLOv10 detection model only detects three foreign objects, and one small target (collapsed region) is missed, as shown in Figure 9c. This missed detection is primarily due to the small size and low contrast of the collapsed region, which leads to insufficient feature activation in the deeper layers of the network. Consequently, the confidence score of the defect falls below the classification threshold, resulting in omission. Moreover, the original YOLOv10 model lacks spatial priors or geometric cues to reinforce defect localization in ambiguous scenarios, making it less robust to subtle or low-visibility targets. In contrast, the improved YOLOv10 detection model correctly detected all four defective regions, as shown in Figure 9d. This shows that the improved algorithm helps to solve the missed detection problem. Moreover, the improved algorithm will give higher confidence scores to the defect regions, which will better accomplish the target detection task for dies.

### 3.3. Comparison of Models and Efficiency

In this study, several commonly used metrics were adopted to evaluate the performance of deep-learning-based detection models, including Precision, *mAP*, Detection Time, and Model Weights. These metrics comprehensively reflect model effectiveness in terms of accuracy, inference speed, and computational efficiency, which are critical for real-time industrial wafer defect inspection. The *mAP* provides an overall measure of detection accuracy, detection time evaluates the real-time performance, and model weights determine the deployment feasibility under limited hardware conditions. To compare the effectiveness of the different algorithms in die defect detection, representative models from the YOLO series—YOLOv5, YOLOv8, YOLOv10, and YOLOv10 + BiFPN—were selected based on their widespread adoption and architectural evolution. These models span from earlier to more advanced YOLO frameworks, providing a balanced and meaningful comparison baseline.

Furthermore, to ensure statistical robustness, each model was trained and evaluated independently five times under identical experimental conditions. The average values and standard deviations of each metric were computed. A paired *t*-test was conducted between the baseline YOLOv10 and the proposed CTM-IYOLOv10 across all metrics. The resulting *p*-values were all below 0.01, confirming that the observed performance improvements are statistically significant and not attributable to random variation. The comparative results are summarized in Table 5.

As presented in Table, the proposed CTM-IYOLOv10 achieves overall performance improvements compared with baseline YOLOv10. The *mAP* increases from 0.978 to 0.981, and the detection time is reduced by 23.2%, from 0.410 s to 0.315 s. Meanwhile, the model size is compressed by 52.3%, from 6.5 MB to 3.1 MB, without loss of precision. To further validate the effectiveness of each improvement, an ablation study was conducted. By introducing the BiFPN structure into YOLOv10, the performance was noticeably improved. On this basis, the integration of the clustering–template matching strategy and lightweight modules such as GhostConv results in additional gains, achieving the best trade-off in CTM-IYOLOv10. These improvements are mainly attributed to the combination of CTM and BiFPN, which enhances the feature representation and reduces the computational redundancy. Compared with earlier versions such as YOLOv5 and YOLOv8, CTM-IYOLOv10 not only achieves higher accuracy but also delivers significantly faster inference and smaller model size, further confirming its advantage in real-time wafer defect detection under resource-constrained conditions.

## 4. Conclusions

In this study, we achieved real-time defect type detection of wafers by capturing wafer images on the bench using an improved template matching algorithm, improved YOLOv10 defect target detection, and precise localization of defect regions after detection. Clustering–template matching is used to improve the efficiency of die detection, solve the problem of redundant results in the original template matching, and successfully locate each complete wafer in the segmented field of view. To improve the YOLOv10 network, the GhostNet idea is introduced and then fused with Conv into a new module, GhostConv, replacing Conv in the backbone part of the original network structure to reduce the amount of parameter computation and effectively reduce the model weight. In the Neck part, YOLOv10 adopts an enhanced BiFPN structure by design, which significantly reduces the feature information loss and improves the recognition accuracy of small targets in die images. In addition, two data enhancement methods, Mosaic and Copy–Paste, were added to the input side to improve the accuracy and robustness of the model.

The above improvements enhance the performance of the wafer defect detection model, resulting in a detection accuracy of 98.1%, while reducing the detection time by 23.2% and the model size by 52.3% compared with the baseline YOLOv10. These metrics were obtained through repeated experiments and statistical validation, demonstrating the robustness of the proposed framework. The improvements in accuracy are particularly critical in industrial wafer inspection, where even minor reductions in false negatives can significantly impact yield. Meanwhile, the reduced model size and inference time support deployment in resource-constrained environments such as edge devices.

These results confirm the suitability of the method for accurate and efficient real-time wafer defect detection, particularly in scenarios with constrained computational resources. However, the method also has limitations. The effectiveness of the clustering–template matching module depends on the regularity and integrity of die layouts, and performance may degrade in scenarios with irregular wafer structures or heavily damaged regions. Future work will explore adaptive strategies to improve robustness under such conditions.

## Figures and Tables

**Table 1:** Comparison of template matching results before and after improvement.

	Number of Matching Results	Actual Number	Redundant Results
Template matching algorithm	8	3	5
Clustering–template matching algorithm	3	3	0

**Table 2:** Dataset Composition.

Defect Type	Stain	Collapse	Cutoff
Number of defects	2085	2125	2096

**Table 3:** Training environment and hardware platform parameters.

Parameters	Configuration
CPU	i7-12700
GPU	NVIDIA GeForce RTX 3060ti
GPU memory size	16G
Deep learning architecture	Pytorch1.9.2 + Cuda11.4 + cudnn11.4

**Table 4:** Key parameters set during model training.

Parameters	Values
Epochs/Step	300
Momentum	0.937
Initial Learning rate	0.01
Weight decay	0.0005
Batch size	32
Input image size/Pixel	640 × 640
Optimizer	SGD
Data enhancement strategy	Mosaic

**Table 5:** Performance comparison of detection algorithms.

	YOLOv5	YOLOv8	YOLOv10	YOLOv10 + BiFPN	CTM-IYOLOv10
Precision	0.946	0.959	0.965	0.970	0.971
*mAP*	0.972	0.975	0.978	0.979	0.981
Detection time (s)	0.610	0.562	0.410	0.395	0.315
Weights (MB)	13.7	7.1	6.5	5.2	3.1

## Data Availability

The data that support the findings of this study are available from the corresponding author upon reasonable request.
